# 136. Prevalence of Plasmid-Mediated Antibiotics Resistance Genes in *Klebsiella pneumoniae* in United States

**DOI:** 10.1093/ofid/ofac492.214

**Published:** 2022-12-15

**Authors:** Munok Hwang, Hosoon Choi, Chetan Jinadatha

**Affiliations:** Central Texas Veterans Health Care System, Temple, Texas; Central Texas Veterans Health Care System, Temple, Texas; Central Texas Veterans Health Care System, Temple, Texas

## Abstract

**Background:**

Multidrug resistant (MDR) bacteria which resist at least three different antibiotic classes are serious threat to public health and patient treatment. Antimicrobial resistant (AMR) genes are often mediated through plasmids due to its horizontal transferability from bacteria to bacteria. Here, we assessed the prevalence of AMR genes in the USA by analyzing longitudinal *K. pneumoniae* plasmid genome data obtained from PATRIC (Pathosystems Resources Integration Center) database.

**Methods:**

The PATRIC database have 176 *K. pneumoniae* plasmid genome data collected in 9 states of US from 2004 to 2019. The isolation source are various patient samples including urine, blood, etc. The sequence information was downloaded from GeneBank. The AMR genes on plasmids of *K. pneumoniae* were identified using open-source AMR database, Resfinder which search AMR genes for 16 types of antibiotic classes.

**Results:**

Multidrug resistance was spread all over the US as most of isolates have AMR genes against more than one antibiotic classes, and some isolates contain against up to 8 antibiotic classes AMR genes. Most common AMR genes are against 9 classes including Aminoglycosides, beta-lactamase (carbapenem), Chloramphenicol, Macrolide, Quinolone, Rifampicin, Sulfonamide, Tetracycline, and Trimethoprim. Aminoglycosides and beta-lactamase including carbapenem resistance genes showed higher frequency than other classes of antibiotics. Carbapenem resistance genes, especially *blaKPC* showed higher frequency in east region of US including NY, PA, and VA (Fig 1A). While some resistances reduced over time, Aminoglycosides and beta-lactamase resistance genes are sharply increased (Fig 1B). Many isolates contain 3∼4 plasmids and plasmid amplicon types were various by isolates but no co-relation between plasmid types and AMR genes was observed.

**Conclusion:**

Database analysis of *K. pneumoniae* isolates from different regions and time provides insight of the prevalence of AMR genes. The prevalence of the multidrug resistance obtained from databases analysis showed the abundance of AMR genes regardless of the region or time, especially for Aminoglycoside and beta-lactamase. Bacterial sequence database such as PATRIC is a useful tool for tracking the existence and emergence of AMR genes.

**Disclosures:**

**Chetan Jinadatha, MD, MPH**, AHRQ R01 Grant-5R01HS025598: Grant/Research Support|EOS Surfaces: Copper Coupons and materials for testing.

**Figure 1A.**
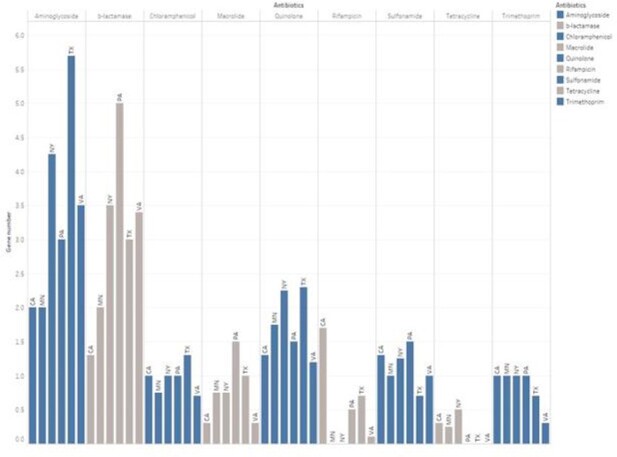
The number of antibiotics resistance genes over the geographical region by the isolates. The states that have more than 3 isolates were included in the analysis.

**Figure IB.**
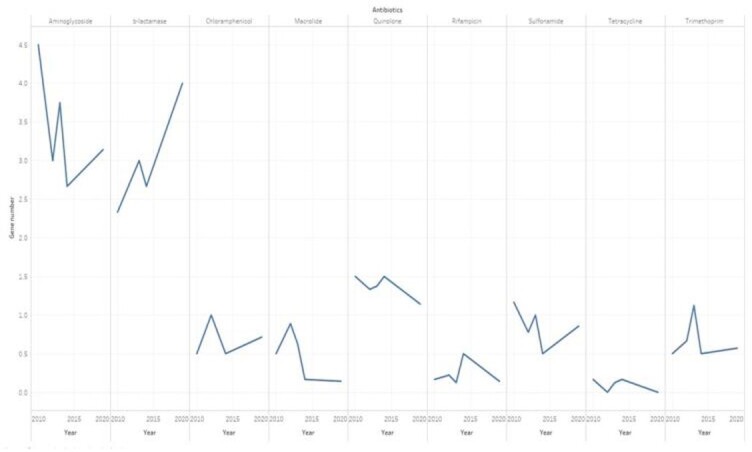
Changes in the number of antibiotics resistance genes over the years. Only time points that have more than 3 isolates been included in the analysis.

